# Renoprotective Effect of Liraglutide Is Mediated via the Inhibition of TGF-Beta 1 in an LLC-PK1 Cell Model of Diabetic Nephropathy

**DOI:** 10.3390/cimb44030072

**Published:** 2022-02-25

**Authors:** Vjera Ninčević, Milorad Zjalić, Tea Omanović Kolarić, Martina Smolić, Tomislav Kizivat, Lucija Kuna, Aleksandar Včev, Ashraf Tabll, Ines Bilić Ćurčić

**Affiliations:** 1 Department of Pharmacology, Faculty of Medicine, Josip Juraj Strossmayer University of Osijek, J. Huttlera 4, 31000 Osijek, Croatia; vnincevic@mefos.hr (V.N.); tomanovic@mefos.hr (T.O.K.); msmolic@mefos.hr (M.S.); lkuna@fdmz.hr (L.K.); 2 Department of Pharmacology and Biochemistry, Faculty of Dental Medicine and Health, Josip Juraj Strossmayer University of Osijek, Crkvena 21, 31000 Osijek, Croatia; 3 Department of Medical Biology and Genetics, Faculty of Medicine, J.J. Strossmayer University of Osijek, J. Huttlera 4, 31000 Osijek, Croatia; mzjalic@mefos.hr; 4 Clinical Institute of Nuclear Medicine and Radiation Protection, University Hospital Osijek, 31000 Osijek, Croatia; tkizivat@mefos.hr; 5 Department for Nuclear Medicine and Oncology, Faculty of Medicine, Josip Juraj Strossmayer University of Osijek, J. Huttlera 4, 31000 Osijek, Croatia; 6 Department of Pathophysiology, Physiology and Immunology, Faculty of Dental Medicine and Health Osijek, J.J. Strossmayer University of Osijek, 31000 Osijek, Croatia; avcev@fdmz.hr; 7 Microbial Biotechnology Department, Genetic Engineering and Biotechnology Research Division, National Research Center, Cairo 12622, Egypt; ashraftabll@yahoo.com; 8 Department of Diabetes, Endocrinology and Metabolism Disorders, University Hospital Osijek, 31000 Osijek, Croatia

**Keywords:** diabetic nephropathy, TGF- beta 1, Liraglutide, LLC-PK1 cells

## Abstract

Background: Recently published research demonstrated direct renoprotective effects of the glucagon-like peptide-1 receptor agonist GLP 1 RA, but the relevant molecular mechanisms are still not clear. The aim of this research was to assess the effects of Liraglutide in a cell culture model of diabetic nephropathy on cell viability, antioxidant (GSH) and transforming growth factor beta 1 (TGF- β1) levels and extracellular matrix (ECM) expression. The metabolic activity in hyperglycemic conditions and the effect of Liraglutide treatment were assessed by measuring Akt, pAkt, GSK3β, pGSK3β, pSTAT3, SOCS3, iNOS and NOX4 protein expression with Western blot. F actin distribution was used to assess the structural changes of the cells upon treatment. Materials and methods: The cells were exposed to high glucose (HG30 mM) followed by 0.5 mM H_2_O_2_ and a combination of glucose and H_2_O_2_ during 24 h. Subsequently, the cells were treated with different combinations of HG30, H_2_O_2_ and Liraglutide. Cell viability was determined by an MTT colorimetric test, and the GSH, TGF-β1 concentration and ECM expression were measured using a spectrophotometric/microplate reader assay and an ELISA kit, respectively. Western blotting was used to detect the protein level of Akt, pAkt, GSK3β, pGSK3β, pSTAT3, SOCS3, iNOS and NOX4. The F-actin cytoskeleton was visualized with Phalloidin stain and subsequently quantified. Results: Cell viability was decreased as well as GSH levels in cells treated with a combination of HG30/H_2_O_2,_ and HG30 alone (*p* < 0.001). The addition of Liraglutide improved the viability in cells treated with HG30, but it did not affect the cell viability in the cell treated with the addition of H_2_O_2_. GSH increased with the addition of Liraglutide in HG30/H_2_O_2_ (*p* < 0.001) treated cells, with no effect in cells treated only with HG30. TGF-β1 levels (*p* < 0.001) were significantly increased in HG30 and HG30/H_2_O_2_. The addition of Liraglutide significantly decreased the TGF-β1 levels (*p* < 0.01; *p* < 0.05) in all treated cells. The synthesis of collagen was significantly increased in HG30/H_2_O_2_ (*p* < 0.001), while the addition of Liraglutide in HG30/H_2_O_2_ significantly decreased collagen (*p* < 0.001). Akt signaling was not significantly affected by treatment. The GSK3b and NOX4 levels were significantly reduced (*p* < 0.01) after the peroxide and glucose treatment, with the observable restoration upon the addition of Liraglutide suggesting an important role of Liraglutide in oxidative status regulation and mitochondrial activity. The treatment with Liraglutide significantly upregulated STAT3 (*p* < 0.01) activity, with no change in SOCS3 indicating a selective regulation of the STAT 3 signaling pathway in glucose and the oxidative overloaded environment. A significant reduction in the distribution of F-actin was observed in cells treated with HG30/H_2_O_2_ (*p* < 0.01). The addition of Liraglutide to HG30-treated cells led to a significant decrease of distribution of F-actin (*p* < 0.001). Conclusion: The protective effect of Liraglutide is mediated through the inhibition of TGF beta, but this effect is dependent on the extent of cellular damage and the type of toxic environment. Based on the WB analysis we have revealed the signaling pathways involved in cytoprotective and cytotoxic effects of the drug itself, and further molecular studies in vitro and vivo are required to elucidate the complexity of the pathophysiological mechanisms of Liraglutide under conditions of hyperglycemia and oxidative stress.

## 1. Introduction

Diabetic nephropathy (DN) is a severe chronic complication of diabetes mellitus type I and II (T1DM and T2DM). DN is the most common cause of renal failure, dialysis and transplantation around the world [1]. Studies have shown that diabetic kidney disease (DKD) can lead to end-stage renal disease in 30–40% of DM patients [2], emphasizing the importance of DKD prevention and the development of new therapeutic options targeting not only glycemic control, but also the pathophysiological processes involved in the onset and progression of chronic complications.

Recently published cardiovascular outcome trials (CVOT) revealed that long acting GLP-1 RAs have significant renoprotective effects which could not be attributed only to the glucose-lowering effects of drugs primarily affecting the glomerular damage pathways [3]. There are several different mechanisms that could be responsible for this beneficial effects of GLP-1 RA. One is the reduction of renal oxidative stress including different molecular pathways, such as the inhibition of NAD(P)H through increased cAMP and PKA production or reducing the activation of TGF-β1 and the connective tissue growth factor (CTGF) accountable for the proliferation of mesangial cells and the extracellular matrix of glomeruli demonstrated in in vitro and in vivo models [4,5]. 

However, a major role of tubular damage in DN is also recognized, along with the traditionally known glomerular injury. Yin W et al. showed that GLP-1 RAs reduced albuminuria and ameliorated kidney tubules and tubulointerstitial lesions in a model with diabetic nephropathy rats [6]. Additionally, C-peptide, which was found to inhibit tubulointerstitial fibrosis [7], was increased by GLP-1 Ras, suggesting a potential mechanism of improving tubulointerstitial and tubular injury in GK rats with diabetic nephropathy [6]. 

The exposure of human proximal tubular cells and cortical fibroblasts to high glucose (HG) concentrations can directly induce cell growth and collagen synthesis, detached of glomerular, hemodynamic or vascular pathology [8]. Some studies have shown that fibronectin generation in response to HG is mediated by the polyol pathway in LLC-PK1 cells [9] and that the flux of glucose in the hexosamine pathway mediates ECM production via the stimulation of TGF-β [10]. Likewise, it promotes oxidative stress and leads, through the P13/Akt (Protein kinase B) pathway, to tubular dysfunction. In addition, the TGF-β receptor acts through the phosphorylation of Smad transcription factors, involved in gene regulation associated with cell differentiation, growth arrest and the epithelial–mesenchymal transition (EMT) [8]. Moreover, TGF-β can induce apoptosis and de-differentiation in epithelial cells and many other different cellular effects, which may promote the progression of diabetic nephropathy [11]. TGF-β’s involvement in the signaling pathways of tubular damage, cell apoptosis and oxidative stress, as well as diabetic nephropathy, may be one of the key mediators of GLP -1 RA renoprotective actions [12].

Akt is a significant mediator of insulin activity and has been established as a fundamental gene necessary for maintaining regular glucose homeostasis [13]. Glycogen synthase kinase-3β (Gsk-3β), a protein downstream of Akt, is broadly associated with the control of mitochondrial functions [14]. Akt is activated by phosphorylation and activated Akt (p-Akt) can conversely control GSK3β. Akt can directly phosphorylate GSK-3β at Ser9, negatively controlling its kinase activity, while the active form of GSK-3β may phosphorylate VDAC1 on threonine 51, leading to reduced HK-II binding to mitochondria. The inactivation of GSK-3β by Akt thereby preserves the integrity of mitochondria [14,15]. 

The signal transducer and transcription activator (STAT) is a family of transcription factors activated by tyrosine phosphorylation via Janus kinases (JAKs) [16]. The members of the STAT family are involved in a number of cell differentiation processes and regulate tissue-specific gene expression [17]. Transcription factors such as the signal transducer and activator of transcription 3 (STAT3) are activated under diabetic conditions [18]. Although STAT3 is mainly prevalent in the cytosol and nucleus, there is growing evidence that it can also be found in mitochondria where it affects cellular respiration [19].

The Janus kinase/signal transducers and activators of the transcription (JAK/STAT) pathway are involved in the progression of DN and are negatively conducted by the suppressors of cytokine signaling (SOCS) proteins. Between the various mechanisms related to JAK/STAT negative regulation, the family of suppressors of cytokine signaling (SOCS) proteins has been suggested as a new goal for a therapeutic approach of DN in humans by regulating the JAK/STAT signaling pathway [20].

NADPH oxidases of the Nox family are the main source of ROS in the diabetic kidney and are crucial mediators of redox signaling in glomerular and tubulointerstitial cells in a diabetic environment. Nicotinamide adenine dinucleotide phosphate oxidase (NOX) is a membrane-bound enzyme responsible for the development of ROS in hyperglycemia. The overexpression of NOX in pathological cases has been linked with the stimulation of aldose reductase, advanced glycosylation products, the hexosamine pathway, protein kinase C and the progression of diabetic injury [21,22].

NOS protein iNOS can be found in different cell types, for example in macrophages, vascular smooth muscle cells and glomerular mesangial cells, producing huge amounts of NO stirred by endotoxins and cytokines. The stimulation of iNOS due to hyperglycemia may lead to the enhanced production of NO, which in turn promotes diabetic hyperfiltration and glomerular aberrations in diabetes [23].

The aim of this study was to investigate the effect of GLP-1 RA Liraglutide on cell viability, oxidative stress and TGF- β levels, in a model of diabetic nephropathy in LLC-PK1 cells. The protein expressions of Akt, pAkt, GSK3β, pGSK3β, pSTAT3, SOCS3, iNOS and NOX4 were also analyzed in order to assess the Liraglutide effects under glucose overload and ROS (reactive oxygen species) stress, given their role in the leptin and insulin signaling pathways, forming a complex network that keeps cells in homeostasis under physiological conditions.

## 2. Materials and Methods

### 2.1. Cell Culture and Treatment

The LLC-PK1 cell line was isolated from a kidney of a healthy, male, Hampshire pig and shows the characteristics of proximal kidney tubules (a gift from Professor Carl Verkoelen’s laboratory at the Urology Clinic of Erasmus Medical Center in Rotterdam, The Netherlands). LLC-PK1 cells were sub-cultivated in Dulbecco’s modified Eagle medium (DMEM) supplemented with 10% fetal bovine serum (FBS/Thermo Fisher Scientific Cat. No. 16000036) and an antibiotic solution (penicillin /streptomycin/Thermo Fisher Scientific Inc., Waltham, MA, USA) at 37 °C. When the cells reached an 80–90% confluence, they were exposed to pathophysiological mediators that mimic DN: high glucose and oxidative stress. At first, the cells were treated with high glucose (D-(+)-Glukoza, bezvodna, pro analysi, Kemika) at different concentrations (1.5, 30 mM), followed by 0.5 mM H_2_O_2_ (Grammol, Zagreb, Croatia, Cat. No. 7722-84-1), and at last with a combination of glucose and H_2_O_2_ (HG30/0.5 mM) for 24 h. Subsequently, the cells were treated with different combinations of glucose and GLP-1 receptor analogue Liraglutide (HG 30 mM/10 nM, HG30/20 nM) and combinations of glucose, H_2_O_2_ and Liraglutide (HG30/H_2_O_2_/10 nM, HG30/H_2_O_2_/20 nM). The cells treated with HG30 and HG30/H_2_O_2_ were compared to control cells; in the second group, the cells treated with HG30 were compared to cells treated with HG30/Lira10 and HG30/Lira20. In the third group, the cells treated with HG30/H_2_O_2_ were compared to cells treated with HG30/H_2_O_2_/Lira10 and HG30/H_2_O_2_/Lira20. The experiments were made in triplicates.

### 2.2. Assessment of Cell Viability

The extent of the damage in the DN proximal tubule and the effect of the drug on cell viability were determined by an MTT colorimetric test. The absorption of each sample was measured at 450 nm on a microplate reader (iMarkTM Microplate Absorbance Reader; Bio-Rad, Hercules, CA, USA) according to the manufacturer’s protocol. The absorbance value from the control group was set as 100%, and the values from the treatment groups were expressed as a percentage of control. 

### 2.3. Assesment of the Oxidative Stress

The oxidative stress of the cells was evaluated by measuring the total glutathione (tGSH) concentration using a spectrophotometric/microplate reader assay method. After incubation, the GSH concentration was determined using a commercially available colorimetric kit according to the manufacturer’s protocol (Glutathione Assay Kit, Signa-Aldrich, Saint Louis, MO, USA). The response was measured with a microplate reader (iMarkTM Microplate Absorbance Reader) at 412 nm. The results were shown in nanomoles per milliliter of sample.

### 2.4. Measurement of TGF-β Levels in an In Vitro Mimic Model of DN in Proximal Tubule Cells

The total TGF-β1 was measured using a Human/Mouse/Rat/Porcine/Canine TGF-beta 1 Quantikine ELISA Kit (Cat No. DB100B) according to the manufacturer’s instructions. On the first day of the experiment, the cells were plated at a density of 1.5 × 10^5^ cells/mL of medium in 6-well plates and treated with different compounds according to the aforementioned protocol. On the third day, the cells were scraped and detached from the cultured dish surface and centrifuged at 140× *g* for 7 min at 4 °C. First, we conducted the sample activation procedure. We added 1 N HCL to 100 uL of cell supernatant and mixed well. This was followed by an incubation at room temperature for 10 min. The acidified sample was then neutralized by adding 20 uL of 1.2 N NaOH/0.5 M HEPES, vortexed for a minimum of 10 s, and an immunoassay procedure was immediately started according to the manufacturer’s instructions.

### 2.5. Measurement of ECM Expression in an In Vitro Mimic Model of DN in Proximal Tubule Cells

LLC-PK1 cells were plated on a 75 cm^2^ Petri dish, grown to 80–90% confluency and treated with different compounds according to the aforementioned protocol. The cells were scraped and detached from the cultured dish surface, transferred to a Falcon tube and centrifuged at 140× *g* for 7 min at 4 °C. The supernatant was discarded, and the pellet (250 uL) was washed with Ammonii acetate (150 mM) and homogenized on ice with an ultrasonic homogenizer Bandelin Sonopuls 2070 (BANDELIN electronic GmbH & Co. KG, Berlin, Germany). The homogenate was centrifuged for 15 min at 1000× *g* at 4 °C. In each tube of homogenized aqueous solution, 750 uL of 25% saturated (NH_4_)_2_SO_4_ was added and incubated overnight at 4 °C under constant agitation. On the next day, the samples were centrifuged at 21,000× *g* 40 min at 4 °C, and collagen was isolated. The supernatant was discarded, and the pellet was solubilized in 1mL of 0.5 M HAc (acetic acid), leaving aliquots of collagen from the culture medium. We transferred 100 uL of collagen aliquots to 2 mL conic tubes, homogenized for 5 s, with a cycle set at 9 and 100% of the power, and precipitated with a 1 mL of 50 uM (69 ug/mL) dye Sirius Red (Sigma-Aldrich Chemical Co., Saint Louis, MO, USA) solution in 0.5 M acetic acid. The samples were vortexed, incubated for 30 min at room temperature to achieve spontaneous precipitation and then centrifuged for 40 min at 21,000× *g*. The supernatant was disposed of, and the pellet was diluted in 1 mL 0.1 N KOH for 15 min at room temperature. The absorbance was measured at a wavelength of 490 nm. 

### 2.6. Protein Extraction and Western Blot Method in the In Vitro Mimic Model of DN in Proximal Tubule Cells

After differentiation in a 75 cm^2^ Petri dish and a growth to a 80–90% confluency, the cells were harvested, transferred to a 1.5 mL Eppendorf tube and pelleted in a centrifuge at 130× *g* for 5 min at 4 °C. The supernatant was separated, followed by the addition of 600 µL of a homogenization buffer. The cells were homogenized on ice with an ultrasonic homogenizer Bandelin Sonopuls 2070 (BANDELIN electronic GmbH & Co. KG, Berlin, Germany), and the homogenate was centrifuged for 15 min at 1000× *g* at 4 °C. The pellet was discarded, and the supernatant was used in further analyses. The supernatant protein content was measured using a Bradford protein assay. Technical triplicates of the cell homogenate sample were pipetted, and 1 µL of the standard sample and 250 µL of Bradford reagent were pipetted to each well. The samples were incubated at room temperature for 15 min and read at the iMark microplate reader (Bio-rad, Hercules, CA, USA) at 595 nm. A dilution of sample aliquots to 1 mg/mL was achieved with a 1× PBS buffer and mixed with a Western blot sample buffer in a 1:5 ratio. The aliquots were heated up to 100 °C for 5 min and stored at 4 °C. The prepared proteins were separated by sodium dodecyl sulfate (SDS)-polyacrylamide gel (12%) electrophoresis in a Hoeffer mighty small electrophoresis system (Hoeffer Inc. San Francisco, CA, USA) with a continuous current of 15 mA per gel. The separated proteins were transferred to polyvinylidene difluoride (PVDF) membranes in a TE22 Mighty small transfer tank (Thermo Fisher Scientific, Waltham, MA, USA). Nonspecific reactions were blocked by a solution of 3% bovine serum albumin (BSA) in a 1× PBS buffer with 0.1% Tween 20 detergent (PBST). The membrane was then incubated in the primary antibody solution overnight on a shaker at +4 °C in the dilutions shown in Table 1. The membranes were washed for 10 min 3 times in a PBST buffer and incubated in a biotin-labeled secondary antibody for 2 h at room temperature (Table 1). The membranes were washed again in a PBST buffer (3 × 10 min) and incubated in a streptavidin-HRP complex for 1 h at room temperature. The washing of secondary antibodies and the streptavidin-HRP complex was carried out 3 times for 10 min in the PBST buffer. Specific proteins were detected using a chemiluminescent detection solution Immobilon ^®^ Forte Western HRP Substrate (Millipore, Burlington, MA, USA) using the ChemiDoc™ Imaging system. Glyceraldehyde 3-phosphate dehydrogenase (GAPDH) was used as an internal control. ImageJ-Fiji software was used to quantitatively analyze the signals of the investigated proteins.

### 2.7. Measurement of Treatment Effectiveness the and Influence on Cell Morphology by Visualizing the F-Actin Cytoskelet with Phalloidin Stain

The cells were maintained and treated according to the previously described protocol. Cell morphology (F-actin cytoskeleton) was visualized with Phalloidin stain (Rhodamine Phalloidin Reagent (ab235138)) according to the manufacturer’s instructions and imaged using an inverted microscope (Axioscop 2 Mot Microscope, Carl Zeiss, Göttingen Germany).

LLC-PK1 cells were grown on glass cover-slips inside a Petri dish to reach a 80–90% confluency and treated according to the previously described protocol. LLC-PK1 cell morphology (F-actin cytoskeleton) was visualized using Phalloidin stain (Rhodamine Phalloidin Reagent (ab235138)) as follows: on the third day, the cell culture medium was aspirated carefully, avoiding cell detachment from the plate surface. The cells were then fixed in 2% formaldehyde (2.5 mL per each well) at 4 °C for 30 min. 0.1% Triton X-100 in PBS 1× was added into the fixed cells for 5 min at room temperature to the permeabilized membrane. The cells were washed 3 times every 10 min in PBS 1×. A conjugate working solution, 250 µL of 1× Phalloidin, was added to each well of fixed cells. The cells were incubated in the dark, at room temperature, for 60 min. The nuclei were counterstained using DAPI (1 µg/mL in methanol). Subsequently, the cells were rinsed 2–3 times with PBS 1× and imaged using an inverted microscope (Axioscop 2 Mot Microscope, Carl Zeiss, Göttingen Germany). Cell images were obtained by using a 40× objective adjusted against negative stain control with mounted and without the post-acquisition enhancement of images. Total and immunopositive nuclei were counted in the ImageJ software using QuantIF ImageJ macro. https://www.mdpi.com/1999-4915/11/2/165/htm (accessed on 8 May 2021).

The statistical analyses were performed using a One-way ANOVA with Post-hoc Tukey HSD; *p*-values of < 0.05 were considered statistically significant. The normality of data distribution was tested with a Shapiro–Wilk test. The homoscedasticity of the groups was tested with Bartletts F test. In both tests, the calculated *p* value was higher than 0.05, indicating the normality of the data distribution and the homoscedasticity between groups, which is a prerequisite for the ANOVA. The statistical program Statistica 12 (Tibco, Palo Alto, CA, USA) was applied to all of these experiments.

## 3. Results

In our study, an LLC-PK1 cell line cultured in normal glucose served as a negative control (NG; 1.5 Mm) , while experimental groups were treated with high glucose (HG; 30 mM), H_2_O_2_ (0.5 mM), a combination of glucose and H_2_O_2_ (HG30/H_2_O_2_), a combination of glucose and Liraglutide (HG 30 mM/10 nM, HG30/20 nM) and a combination of glucose, H_2_O_2_ and Liraglutide (HG30/H_2_O_2_/10 nM, HG30/H_2_O_2_/20 nM) for 24 h.

### 3.1. Cell Viability in the LLC-PK1 Cell Line

**Figure 1:**
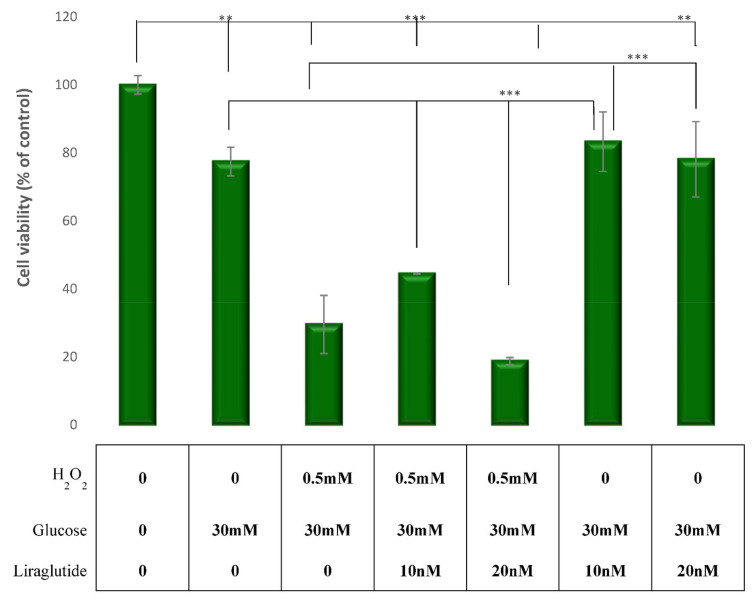
Cell viability assessed by an MTT assay in the experimental LLC-PK1-treated cell lines (control vs. HG30 and HG30/H_2_O_2_; HG30 vs. HG30/Lira10 and HG30/Lira20; HG30/H_2_O_2_ vs. HG30/H_2_O_2_/Lira10 and HG30/H_2_O_2_/Lira20). MTT measurements were done by spectrophotometry at 595 nm. One-way ANOVA _F(8,44) = 67.66, *p* = 9.26 × 10_^−19^ with Tukey HSD post hoc test; ** *p* < 0.01 *** *p* < 0.001. The data are shown as the means ± SD (standard deviation) from three independent biological replicates. Values underneath the x axis represent the amount of compound added in the experiment, and 0 represents that the compound was not added. MTT assay, pig proximal tubule cell line (LLC-PK1), HG (1.5 mM, 30 mM), H_2_O_2_ (0.5 mM), Liraglutide (10 nM, 20 nM).

In the cells grown in hyperglycemia, induced cytotoxicity was observed as the cell viability was decreased. Cells treated with a combination of HG30/H_2_O_2,_ and HG30 alone had a significant decrease in MTT levels compared with controls (*p* < 0.001). Cell viability was improved with the addition of Liraglutide 10 nM to cells treated with HG30, while 20 nM had no effect. However, cell viability was significantly decreased in all experimental groups treated with H_2_O_2_ regardless of Liraglutide addition, as shown in Figure 1.

### 3.2. Measurement of Cellular Glutathione (GSH) Concentration in the LLC-PK1 Cell Line

**Figure 2:**
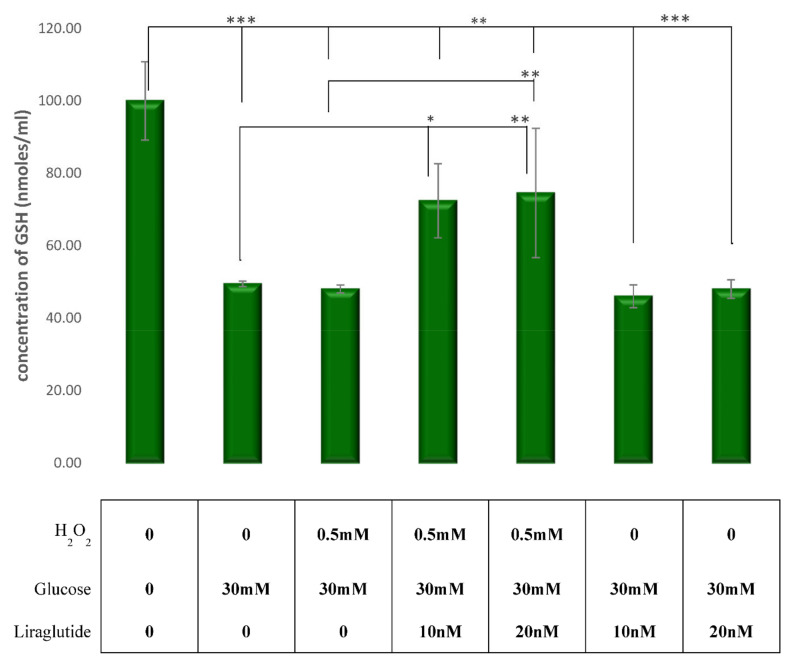
GSH levels in the experimental LLC-PK1-treated cell lines (control vs. HG30 and HG30/H_2_O_2_; HG30 vs. HG30/Lira10 and HG30/Lira20; HG30/H_2_O_2_ vs. HG30/H_2_O_2_/Lira10 and HG30/H_2_O_2_/Lira20). GSH measurements were done by spectrophotometry at 415 nm. One-way ANOVA _F(16,70) = 43.68, *p* = 4.137 × 10_^−25^ with Tukey HSD post hoc test; The values are represented in micromole per milliliter as average. Bars assigned with asterisks are statistically significantly different * *p* < 0.05, ** *p* < 0.01 *** *p* < 0.001. Values underneath the x axis represent the amount of compound added in the experiment, and 0 represents that the compound was not added. The data are shown as the means ± SD (standard deviation) from three independent biological replicates. Cellular glutathione (GSH), pig proximal tubule cell line (LLC-PK1), HG (1.5 mM, 30 mM), H_2_O_2_ (0.5 mM), Liraglutide (10 nM, 20 nM).

GSH levels were measured in the LLC-PK1-treated cell cultures and the control group as shown in Figure 2. There was a significant decrease in GSH levels in cells treated with HG30 and HG30/H_2_O_2_ compared to the untreated controls (*p* < 0.001), as expected. A significant increase of GSH was observed with the addition of Liraglutide 10 and 20 nM in cells treated with HG30/H_2_O_2_ (*p* < 0.01). However, the addition of Liraglutide (both 10 and 20 nM) to HG30 revealed that the cells had no effect on the GSH levels. 

### 3.3. Effects of Glucose, H_2_O_2_ and Liraglutide on TGF-β1 Levels

**Figure 3:**
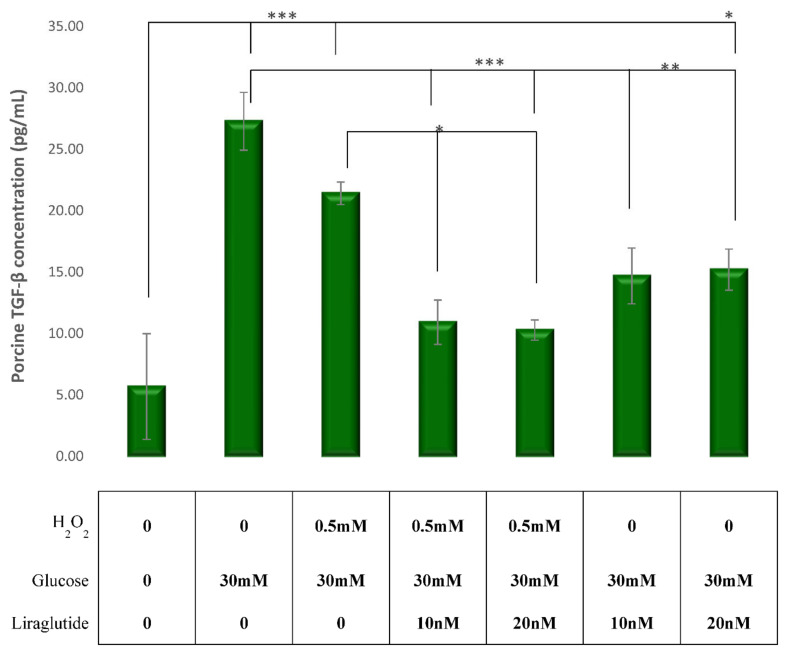
Levels of TGF-β concentrations in the experimental LLCPK1-treated cell lines (control vs. HG30 and HG30/H_2_O_2_; HG30 vs. HG30/Lira10 and HG30/Lira20; HG30/H_2_O_2_ vs. HG30/H_2_O_2_/Lira10 and HG30/H_2_O_2_/Lira20). TGF-β measurements were done by spectrophotometry at 450 nm. One-way ANOVA _F(7,23) = 14.54, *p* = 7.47 × 10_^−6^ with Tukey HSD post hoc test; * *p* < 0.05, ** *p* < 0.01, *** *p* < 0.001. The values are represented in picogram per milliliter as average. Values underneath the x axis represent the amount of compound added in the experiment, and 0 represents that the compound was not added. The data are shown as the means ± SD (standard deviation) from three independent biological replicates. TGF-β Elisa, pig proximal tubule cell line (LLC-PK1), HG (1.5 mM, 30 mM), H_2_O_2_ (0.5 mM), Liraglutide (10 nM, 20 nM).

TGF-β1 levels were significantly increased in HG30- and HG30/H_2_O_2_-treated cells compared to the untreated controls (*p* < 0.001). The addition of Liraglutide in both concentrations significantly decreased TGF-β1 levels in both HG30- (*p* < 0.01) and HG30/H_2_O_2_-treated (*p* < 0.05) cells. The results are shown in Figure 3.

### 3.4. Measurement of ECM Expression in a Cell Culture Model of Diabetic Nephropathy

**Figure 4:**
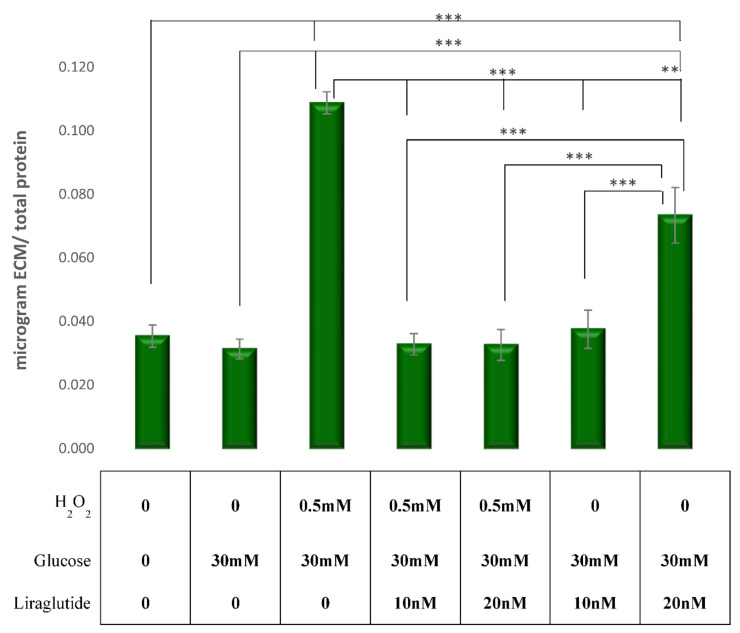
Levels of collagen synthesis in the experimental LLC-PK1-treated cell lines (control vs. HG30 and HG30/H_2_O_2_; HG30 vs. HG30/Lira10 and HG30/Lira20; HG30/H_2_O_2_ vs. HG30/H_2_O_2_/Lira10 and HG30/H_2_O_2_/Lira20). ECM measurements were done by spectrophotometry at 595 nm. One-way ANOVA _F(9,26) = 49.73, *p* = 1.6 × 10_^−6^ with Tukey HSD post hoc test; ** *p* < 0.01, *** *p* < 0.001. The values are represented in microgram ECM per total protein as average. Values underneath the x axis represent the amount of compound added in the experiment, and 0 represents that the compound was not added. The data are shown as the means ± SD (standard deviation) from three independent biological replicates. ECM measurement, pig proximal tubule cell line (LLC-PK1), HG (1.5 mM, 30 mM), H_2_O_2_ (0.5 mM), Liraglutide (10 nM, 20 nM).

The synthesis of collagen was significantly increased in HG30/H_2_O_2_-treated (*p* < 0.001) cells when compared to controls, while the addition of Liraglutide in both concentrations (10 nM and 20 nM) significantly decreased the collagen concentrations compared to HG30/H_2_O_2_-treated cells (*p* < 0.001 for all). The treatment with HG30 did not change the synthesis of collagen compared to controls. The addition of Liraglutide 20 nM in cells treated with HG30 only significantly increased the collagen synthesis compared to cells treated with HG30 only (*p* < 0.01), while the same effect was observed with the addition of 10 nM of Liraglutide. However, the difference was not statistically significant, as shown in Figure 4. 

### 3.5. Protein Extraction and Western Blot Method in a Cell Culture Model of Diabetic Nephropathy

The effects of glucose at different concentrations (1.5, 30 mM) and the combinations of glucose and H_2_O_2_ (30/0.5 mM), of glucose and Liraglutide (30 mM/10 nM, 30/20 nM) and of glucose, H_2_O_2_ and Liraglutide (30/0.5 mM/10 nM, 30/0.5 mM/20 nM) on the protein expression were examined using a Western blot analysis. 

**Figure 5:**
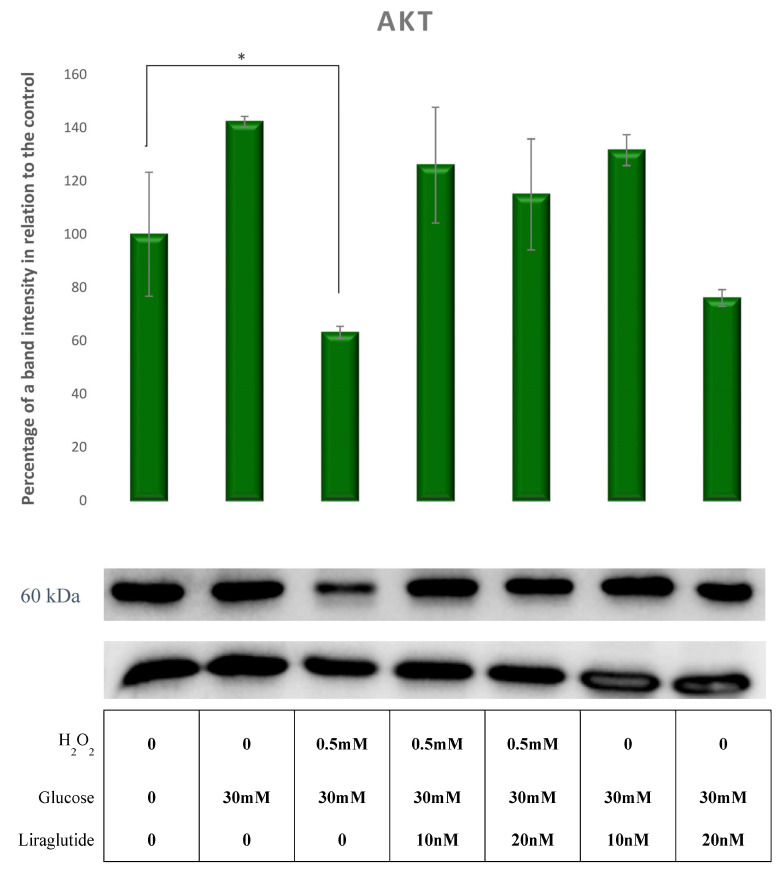
Protein expression in an LLC-PK1-treated cell line (control vs. HG30 and HG30/H_2_O_2_; HG30 vs. HG30/Lira10 and HG30/Lira 20; HG30/H_2_O_2_ vs. HG30/H_2_O_2_/Lira10 and HG30/H_2_O_2_/Lira 20) determined by Western blot. Glyceraldehyde 3-phosphate dehydrogenase (GAPDH) was used as an internal control. The data are shown as the means ± SD (standard deviation) from three independent biological replicates. One-way ANOVA _F(6,20) = 4.04, *p* = 1.47 × 10_^−2^ with Tukey HSD post hoc test; * *p* < 0.05; Values underneath the x axis represent the amount of compound added in the experiment, and 0 represents that the compound was not added. Protein expression, pig proximal tubule cell line (LLC-PK1), HG (1.5 mM, 30 mM), H_2_O_2_ (0.5 mM), Liraglutide (10 nM, 20 nM).

A significant decrease of AKT levels compared to controls was observed in HG30/H_2_O_2_-treated cells (*p* < 0.05). The addition of Liraglutide 10 nM and 20 nM caused an increase in the AKT protein levels relative to HG30/H202-treated cells (*p* = NS), as shown in Figure 5.

**Figure 6:**
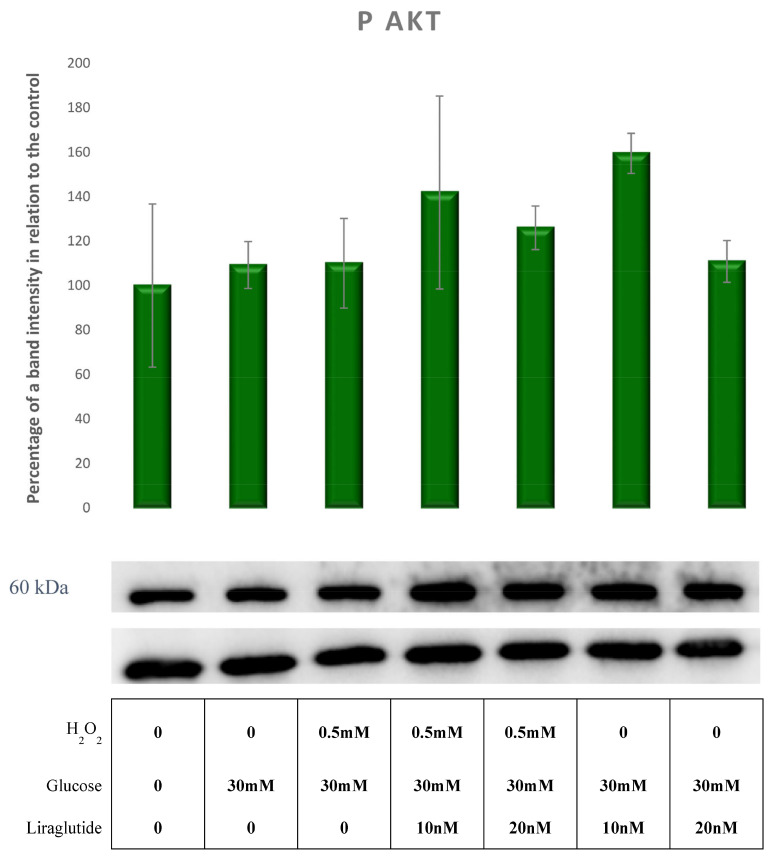
Protein expression in LLC-PK1-treated cell line (control vs. HG30 and HG30/H_2_O_2_; HG30 vs. HG30/Lira10 and HG30/Lira 20; HG30/H_2_O_2_ vs. HG30/H_2_O_2_/Lira10 and HG30/H_2_O_2_/Lira 20) determined by Western blot. Glyceraldehyde 3-phosphate dehydrogenase (GAPDH) was used as an internal control. The data are shown as the means ± SD (standard deviation) from three independent biological replicates. One-way ANOVA _F(6,20) = 0.7921, *p* = 5.91 × 10_^−1^ with Tukey HSD post hoc test. Values underneath the x axis represent the amount of compound added in the experiment, and 0 represents that the compound was not added. Protein expression, pig proximal tubule cell line (LLC-PK1), HG (1.5 mM, 30 mM), H_2_O_2_ (0.5 mM), Liraglutide (10 nM, 20 nM).

Compared to controls, there was no significant change in pAKT expression across all experimental groups, as shown in Figure 6.

**Figure 7:**
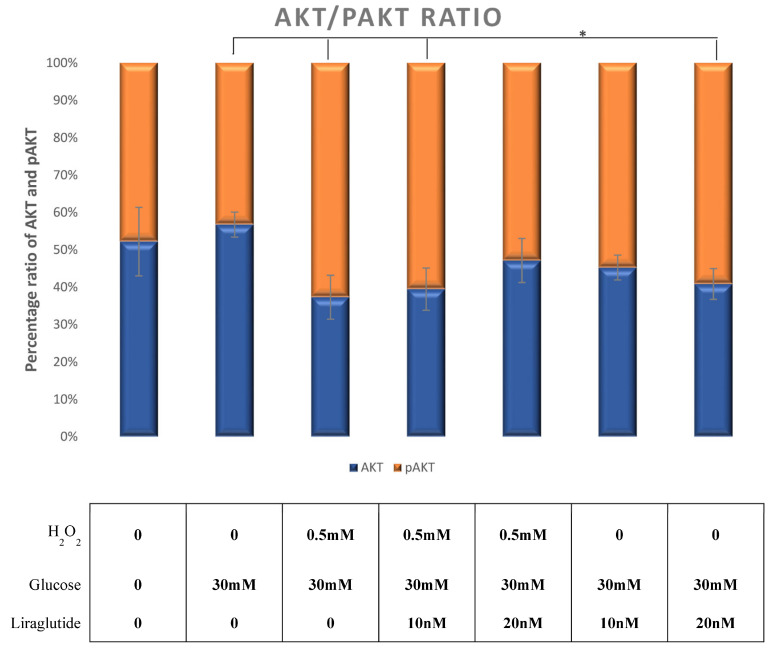
Ratio of pAKT/AKT molecules calculated from protein expression in the LLC-PK1-treated cell line (control vs. HG30 and HG30/H_2_O_2_; HG30 vs. HG30/Lira10 and HG30/Lira 20; HG30/H_2_O_2_ vs. HG30/H_2_O_2_/Lira10 and HG30/H_2_O_2_/Lira 20) determined by Western blot. The data are shown as the means ± SD (standard deviation) from three independent biological replicates. One-way ANOVA _F(6,20) = 4.659, *p* = 8.34 × 10_^−3^ with Tukey HSD post hoc test; * *p* < 0.05. Values underneath the x axis represent the amount of compound added in the experiment, and 0 represents that the compound was not added. Protein expression, pig proximal tubule cell line (LLC-PK1), HG (1.5 mM, 30 mM), H_2_O_2_ (0.5 mM), Liraglutide (10 nM, 20 nM).

The ratio of pAKT/AKT increased as a response to the combined glucose and peroxide treatment. An additional treatment of glucose/peroxide group with 10 nM of liraglutide had no effect on the reduction of the pAKT/AKT ratio. In the peroxide/glucose group additionally treated with 20 nM of Liraglutide, the ratio of pAKT/AKT returned to levels similar to those observed in a glucose-treated group. The induced oxidative damage only by a high glucose treatment with 20 nM of Liraglutide demonstrated an effect of the increased pAKT/AKT ratio similar to the one observed in the peroxide/glucose group, as shown in Figure 7.

**Figure 8:**
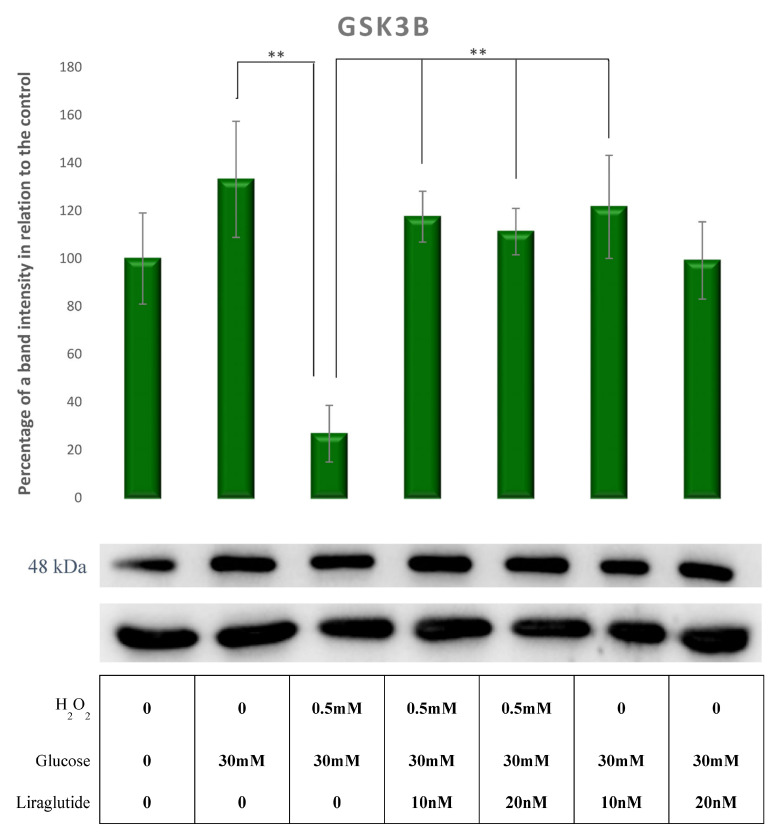
Protein expression in the LLC-PK1-treated cell line (control vs. HG30 and HG30/H_2_O_2_; HG30 vs. HG30/Lira10 and HG30/Lira 20; HG30/H_2_O_2_ vs. HG30/H_2_O_2_/Lira10 and HG30/H_2_O_2_/Lira 20) determined by Western blot. Glyceraldehyde 3-phosphate dehydrogenase (GAPDH) was used as an internal control. The data are shown as the means ± SD (standard deviation) from three independent biological replicates. One-way ANOVA _F(6,20) = 4.23, *p* = 1.23 × 10_^−2^ with Tukey HSD post hoc test; ** *p* < 0.01; Values underneath the x axis represent the amount of compound added in the experiment, and 0 represents that the compound was not added. Protein expression, pig proximal tubule cell line (LLC-PK1), HG (1.5 mM, 30 mM), H_2_O_2_ (0.5 mM), Liraglutide (10 nM, 20 nM).

A decrease in GSK3 β protein levels was demonstrated in HG30/H_2_O_2_-treated cells compared to those treated only with HG30. GSK3 β protein levels were increased in cells treated with HG30/H_2_O_2_/Lira10 and 20 compared to those treated with HG30/H_2_O_2_ (*p* < 0.01), as shown in Figure 8. 

**Figure 9:**
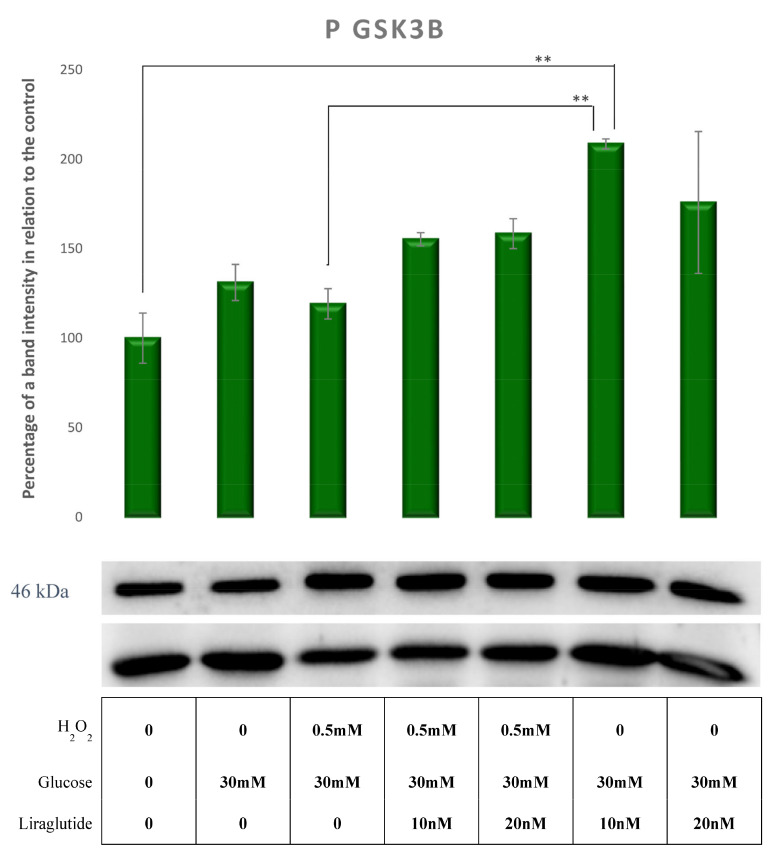
Protein expression in the LLC-PK1-treated cell line (control vs. HG30 and HG30/H_2_O_2_; HG30 vs. HG30/Lira10 and HG30/Lira 20; HG30/H_2_O_2_ vs. HG30/H_2_O_2_/Lira10 and HG30/H_2_O_2_/Lira 20) determined by Western blot. Glyceraldehyde 3-phosphate dehydrogenase (GAPDH) was used as an internal control. The data are shown as the means ± SD (standard deviation) from three independent biological replicates. One-way ANOVA _F(6,20) = 4.589, *p* = 8.86 × 10_^−3^ with Tukey HSD post hoc test; ** *p* < 0.01; Values underneath the x axis represent the amount of compound added in the experiment, and 0 represents that the compound was not added. Protein expression, pig proximal tubule cell line (LLC-PK1), HG (1.5 mM, 30 mM), H_2_O_2_ (0.5 mM), Liraglutide (10 nM, 20 nM).

pGSK3 β protein levels remained unaffected in cells treated with HG30 and HG30/H_2_O_2_ compared to the controls. The addition of both concentrations of Liraglutide demonstrated a tendency to increase pGSK3 β. However, it was not statistically significant, except when comparing HG30/Lira10 with HG30/H_2_O_2_, as shown in Figure 9.

**Figure 10:**
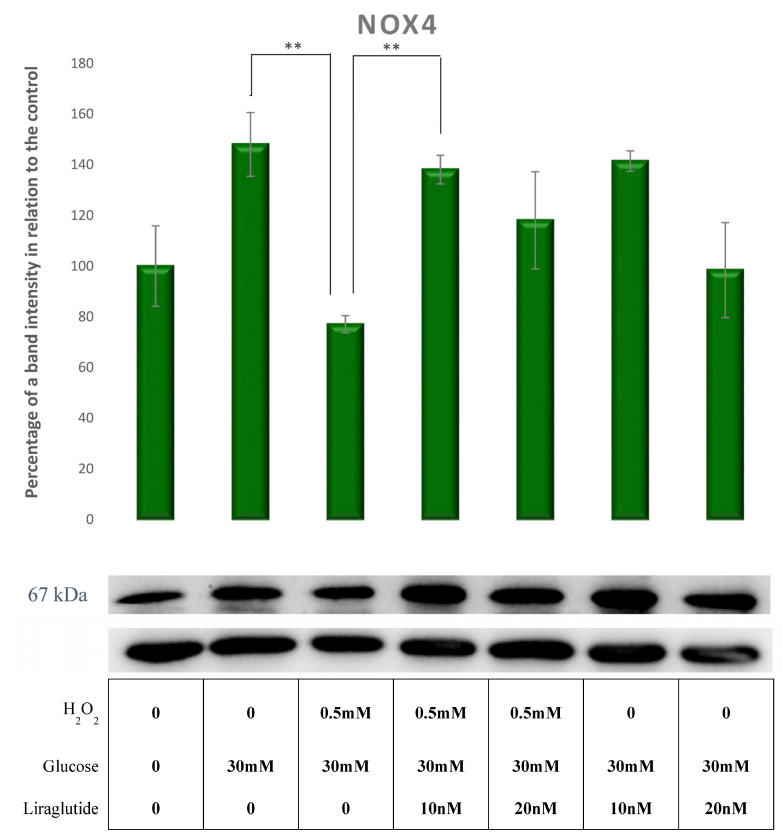
Protein expression in the LLC-PK1-treated cell line (control vs. HG30 and HG30/H_2_O_2_; HG30 vs. HG30/Lira10 and HG30/Lira 20; HG30/H_2_O_2_ vs. HG30/H_2_O_2_/Lira10 and HG30/H_2_O_2_/Lira 20) determined by Western blot. Glyceraldehyde 3-phosphate dehydrogenase (GAPDH) was used as an internal control. The data are shown as the means ± SD (standard deviation) from three independent biological replicates. One-way ANOVA _F(6,20) = 4.154, *p* = 1.32 × 10_^−2^ with Tukey HSD post hoc test; ** *p* < 0.01. Values underneath the x axis represent the amount of compound added in the experiment, and 0 represents that the compound was not added. Protein expression, pig proximal tubule cell line (LLC-PK1), HG (1.5 mM, 30 mM), H_2_O_2_ (0.5 mM), Liraglutide (10 nM, 20 nM).

NOX4 protein levels were not changed by the addition HG30 and HG30/H_2_O_2_ compared to controls, but the cells’ exposure to HG30/H_2_O_2_ caused a significant decrease of NOX4 compared to cells treated only with HG30 (*p* < 0.01). NOX4 protein levels were significantly increased in cells treated with HG30/H_2_O_2_/Lira10 compared to those treated with HG30/H_2_O_2_ (*p* < 0.01), as shown in Figure 10. 

**Figure 11:**
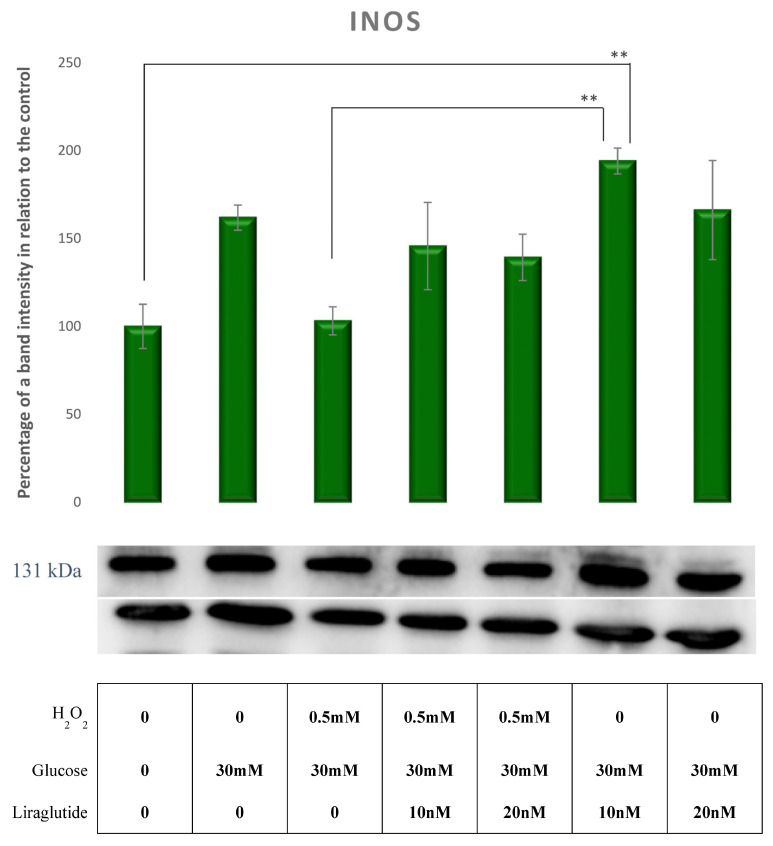
Protein expression in the LLC-PK1-treated cell line (control vs. HG30 and HG30/H_2_O_2_; HG30 vs. HG30/Lira10 and HG30/Lira 20; HG30/H_2_O_2_ vs. HG30/H_2_O_2_/Lira10 and HG30/H_2_O_2_/Lira 20) determined by Western blot. Glyceraldehyde 3-phosphate dehydrogenase (GAPDH) was used as an internal control. The data are shown as the means ± SD (standard deviation) from three independent biological replicates. One-way ANOVA _F(6,20) = 4.241, *p* = 1.218 × 10_^−2^ with Tukey HSD post hoc test; ** *p* < 0.01. Values underneath the x axis represent the amount of compound added in the experiment, and 0 represents that the compound was not added. Protein expression, pig proximal tubule cell line (LLC-PK1), HG (1.5 mM, 30 mM), H_2_O_2_ (0.5 mM), Liraglutide (10 nM, 20 nM).

iNOS levels remained unaffected by the treatment with HG30 and HG30/H_2_O_2_. Cells treated with HG30/Lira10 showed a significant increase of iNOS levels compared to HG30/H_2_O_2_-treated cells and controls, as shown in Figure 11.

**Figure 12:**
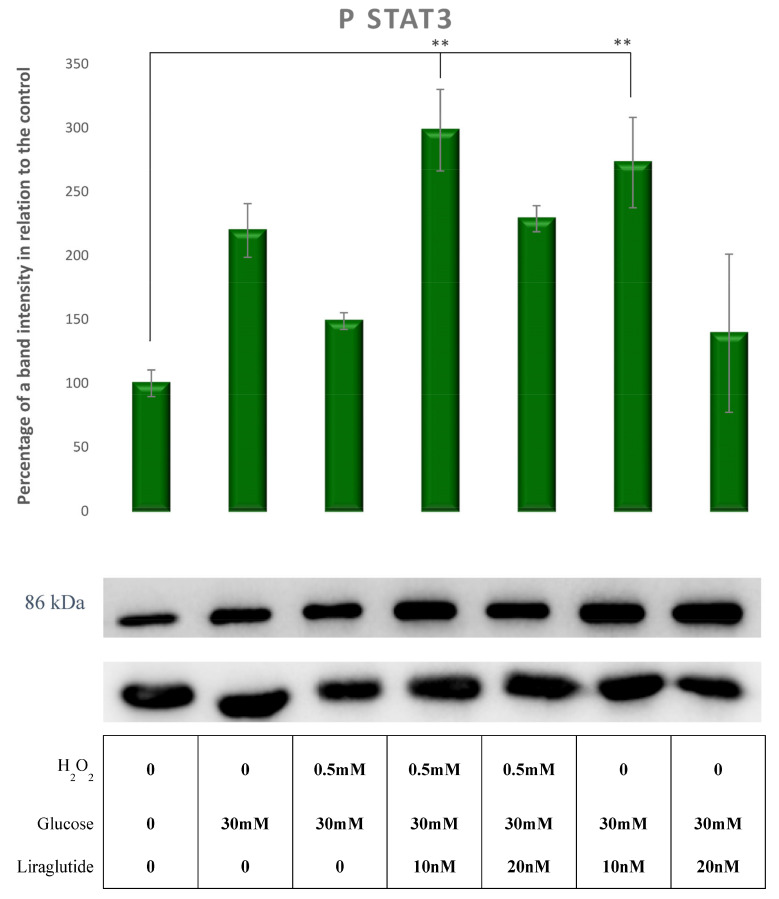
Protein expression in the LLC-PK1-treated cell line (control vs. HG30 and HG30/H_2_O_2_; HG30 vs. HG30/Lira10 and HG30/Lira 20; HG30/H_2_O_2_ vs. HG30/H_2_O_2_/Lira10 and HG30/H_2_O_2_/Lira 20) determined by Western blot. Glyceraldehyde 3-phosphate dehydrogenase (GAPDH) was used as an internal control. The data are shown as the means ± SD (standard deviation) from three independent biological replicates. One-way ANOVA _F(6,20) = 5.57, *p* = 3.88 × 10_^−3^ with Tukey HSD post hoc test; ** *p* < 0.01; Values underneath the x axis represent the amount of compound added in the experiment, and 0 represents that the compound was not added. Protein expression, pig proximal tubule cell line (LLC-PK1), HG (1.5 mM, 30 mM), H_2_O_2_ (0.5 mM), Liraglutide (10 nM, 20 nM).

The protein levels of pSTAT3 in HG30- and HG30/H_2_O_2_-treated cells increased relative to the control group (*p* = NS). The treatment with HG30/H_2_O_2_/Lira10 significantly increased the pSTAT3 levels compared to controls (*p* < 0.01), as shown in Figure 12.

**Figure 13:**
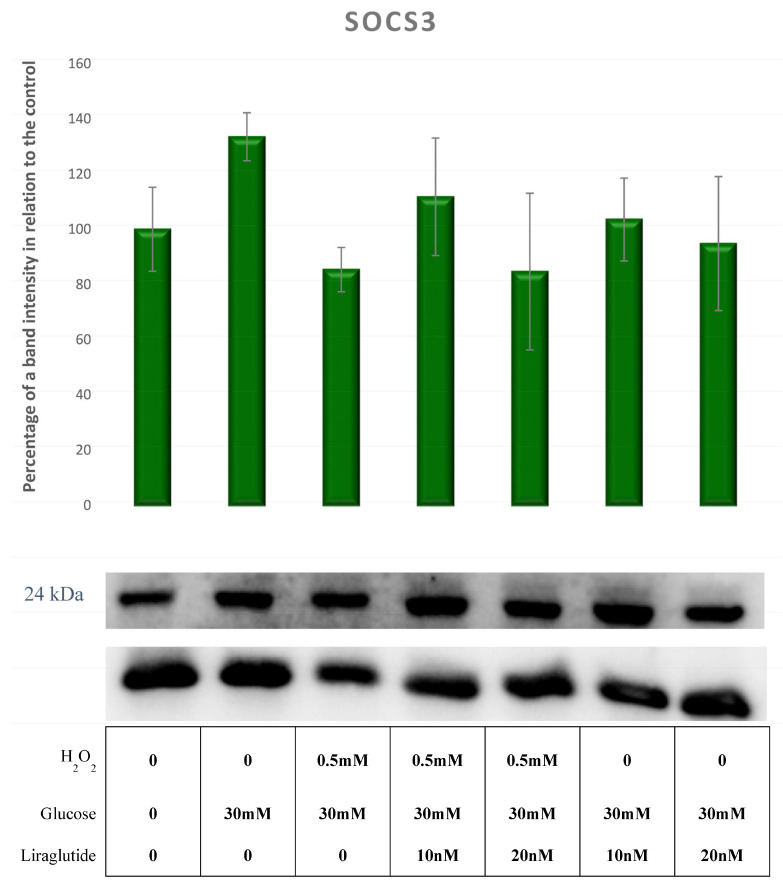
Protein expression in the LLC-PK1-treated cell line (control vs. HG30 and HG30/H_2_O_2_; HG30 vs. HG30/Lira10 and HG30/Lira 20; HG30/H_2_O_2_ vs. HG30/H_2_O_2_/Lira10 and HG30/H_2_O_2_/Lira 20) determined by Western blot. Glyceraldehyde 3-phosphate dehydrogenase (GAPDH) was used as an internal control. The data are shown as the means ± SD (standard deviation) from three independent biological replicates. One-way ANOVA _F(6,20) = 0.819, *p* = 5.73 × 10_^−1^ with Tukey HSD post hoc test. Values underneath the x axis represent the amount of compound added in the experiment, and 0 represents that the compound was not added. Protein expression, pig proximal tubule cell line (LLC-PK1), HG (1.5 mM, 30 mM), H_2_O_2_ (0.5 mM), Liraglutide (10 nM, 20 nM).

There was no significant change in SOCS3 expression across all experimental groups, as shown in Figure 13.

### 3.6. Visualization and Quantification of the F-Actin Cytoskelet with Phalloidin Stain

**Figure 14:**
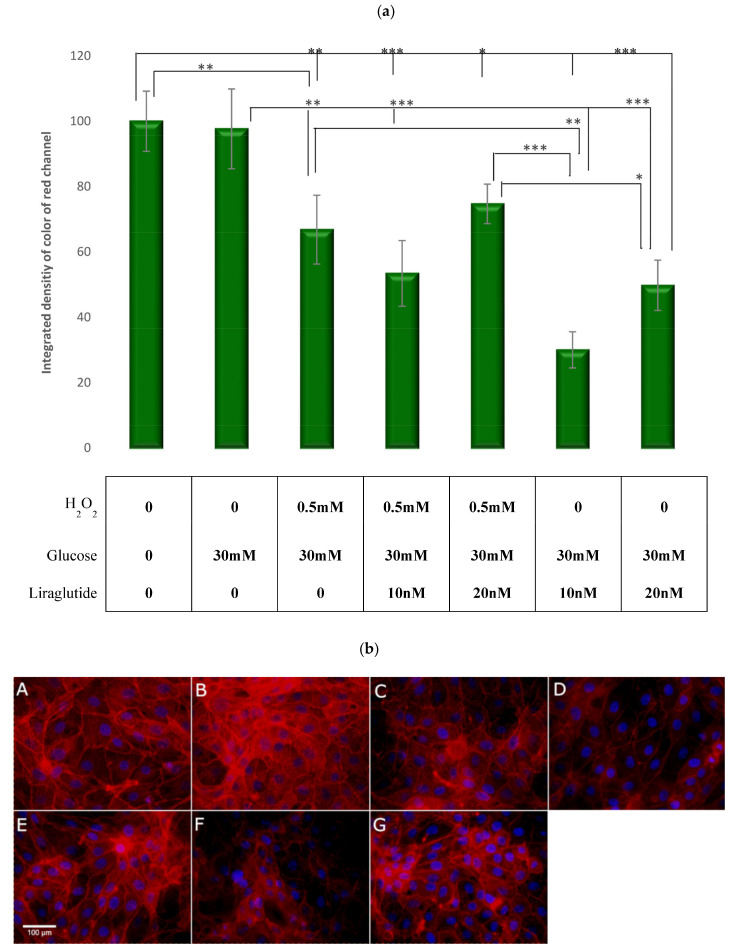
(**a**) Quantification and Visualization of the F-actin cytoskeleton with Rhodamine Phalloidin stain. Levels of total F actin stained by Phalloidin-Rhodamine in the LLC-PK1-treated cell lines (control vs. HG30 and HG30/H_2_O_2_; HG30 vs. HG30/Lira10 and HG30/Lira20; HG30/H_2_O_2_ vs. HG30/H_2_O_2_/Lira10 and HG30/H_2_O_2_/Lira20). Data represent the integrated density of the red color of stained actin per single cell. A higher number equals a more intense stain. One-way ANOVA _F(8,26) = 22.41, *p* = 7.46 × 10_^−8^ with Tukey HSD post hoc test; * *p* < 0.05, ** *p* < 0.01, *** *p* < 0.001. The data are shown as the means ± SD (standard deviation) from three independent biological replicates. Bars assigned with asterisks are statically significantly different. Values underneath the x axis represent the amount of compound added in the experiment, and 0 represents that the compound was not added. Protein expression, pig proximal tubule cell line (LLC-PK1), HG (1.5 mM, 30 mM), H_2_O_2_ (0.5 mM), Liraglutide (10 nM, 20 nM). (**b**) LLC-PK1 cells were labeled for F-actin using Phalloidin labelled with rhodamine and nuclei stained with DAPI. (**A**)—DMEM (control), (**B**)—glucose (HG 30 mM), (**C**)—glucose and H_2_O_2_ (HG/0.5 mM), (**D**)—glucose, H_2_O_2_ and Liraglutide (HG/0.5 mM/10 nM), (**E**)—glucose, H_2_O_2_ and Liraglutide (HG/0.5 mM/20 nM), (**F**)—glucose and Liraglutide (HG30/10 nM), (**G**)—glucose and Liraglutide (HG30/20 nM). The size bar represents 100 µm.

A significant reduction in the distribution of F-actin was observed in cells treated with HG30/H_2_O_2_ (*p* < 0.01), yet no difference was present in HG30-treated cells compared to controls, as shown in Figure 14a,b. The addition of Liraglutide to HG30 led to a significant decrease in the distribution of F-actin (*p* < 0.001), with no effect on cells cultured with HG30/H_2_O_2_. 

## 4. Discussion

The key extracellular conditions in diabetic nephropathy are hyperglycemia, proteinuria, hypoxia and inflammation, and contribute to proximal tubular damage by shifting the hormone-induced release of cytokines (TGF-β) and promoting oxidative stress [24,25,26,27]. So far, in vitro and in vivo findings suggested that the GLP-1 receptor agonist Liraglutide decreases hyperglycemia-induced kidney damage by reducing the proliferation of mesangial cells, albuminuria, oxidative stress and inflammatory cytokines [28,29,30] possibly through the alteration of TGF-β expression, but the molecular mechanisms involved in this process have yet to be fully clarified. 

In our study, treating proximal tubular cells with both HG30 and H_2_O_2_ caused a decrease in cell viability. In a study by Tong et al., a decrease in cell viability and an increase in cell apoptosis in a NRK-52E cell culture were also demonstrated. On the other hand, the treatment with Liraglutide led to an improved viability in the hyperglycemic conditions measured by the MTT levels. Similarly, Zhao et al. proved that Liraglutide improved cell viability in HK-2 cells (human proximal tubular cells) by downregulating caspase-3 expression. Furthermore, upon treatment with H_2_O_2_, Liraglutide lost its protective effect due to extensive oxidative damage that in turn most likely caused cell apoptosis.

Under physiological conditions, cells are equipped with numerous antioxidant systems, including non-enzymatic ones, such as GSH, to limit the effects of free radicals. To a certain level, GSH can control the damage caused by free radicals, but at one point hyper saturation occurs, cells are confronted with oxidative stress, and the addition of external agents is necessary to maintain cell viability, as was shown in a rat model of nephrolithiasis [31]. Our data regarding GSH levels clearly demonstrated that the treatment with HG30 and H_2_O_2_ increased oxidative stress, while the addition of Liraglutide had the beneficial effect of reducing oxidative stress. However, the addition of Liraglutide to cells damaged only by hyperglycemia had no effect, indicating that the protective effect of the drug is achieved predominantly through the prevention of oxidative stress in viable cells before cell apoptosis occurs. Previous research supports this hypothesis. The reduction of oxidative stress by Liraglutide was shown in patients with type-2 diabetes independent of glucose-lowering effect [32]. Furthermore, in a study performed by Yin et al., a recombinant human GLP-1 inhibited protein kinase C (PKC)-β but increased protein kinase A (PKA), reducing oxidative stress in both glomeruli and tubules in diabetic STZ-induced rats [6]. A study by Krause et al. demonstrated that Liraglutide significantly improved GSH levels but had no effect on the reactive oxygen species production [33]. Therefore, we could hypothesize that Liraglutide could have a protective effect on the cell membrane when exposed to free radicals mediated, at least in part, by GSH.

Several studies have suggested that intrinsic renal cells are able to produce inflammatory cytokines and growth factors such as TGF-β1, responsible for the progression of DN [34]. As a fibrogenic cytokine, TGF-β1 is considered a key mediator in DN, as was demonstrated in a large meta-analysis including type-2 diabetic patients with albuminuria [35]. In addition, oxidative stress through the P13K/Akt and JNK pathways leads to the activation of NF-кB, upregulating the expression of TGF-β1 and the connective tissue growth factor [36,37]. The results of our study are in agreement with previous research demonstrating a significant increase of TGF-β1 levels in injured cells, both with hyperglycemia and oxidative stress. In a recently published study, oxidative stress in normotensive rats fed with L-arginin induced renal injury and was mediated by increased NOX4 production [38]. 

Furthermore, Liraglutide led to a decrease of TGF-β1 levels when added to cells exposed to hyperglycemia and/or oxidative stress, suggesting that the protective effect of Liraglutide was mediated through the inhibition of TGF-β1 expression in this model of diabetic nephropathy. Likewise, previous research demonstrated the protective role of Liraglutide, by diminishing renal fibrosis induced by TGF-β1 in NRK-52E cells. The proposed pathophysiological mechanism could be the inhibition of the activation of the TGF-β1/Smad3 and ERK1/2 signaling pathways. Some studies have shown that fibronectin generation in response to HG is mediated by the polyol pathway in LLC-PK1 cells [39] and that the flux of glucose in the hexosamine pathway mediates ECM production via the stimulation of TGF-β [9]. In addition, it promotes oxidative stress and leads, through the P13/Akt pathway, to tubular dysfunction. Hypoxia increases collagen production and TIMP1 expression and decreases MMP2 activity (ECM accumulation) [10,36]. In our study, H_2_0_2_ may have induced ECM expression by a direct or an indirect effect, through the activation of the TGF-β system [40]. A study by Huang L et al. showed that Liraglutide reduced the accumulation of glomerular ECM and renal injury in DN by upregulating the signaling of the Wnt/β-catenin pathway and ameliorated renal injury in diabetic nephropathy. This is in agreement with our results, since an increased amount of collagen was observed in the group treated with high glucose and H_2_O_2_, while a decrease of collagen concentration was noted in groups treated with both concentrations of Liraglutide.

Akt is part of the insulin signaling pathway, and phosphorylation determines Akt activity. The Akt protein levels were minimally altered in all groups except in cells treated with HG30/H_2_O_2_. There was no significant change in the Akt activity with the addition of Liraglutide, indicating that the treatment with Liraglutide was not causing pathological hyperactivation or sub-activation in one important part of the insulin pathway, where the phosphorylation of a significant number of transcription factors occurs via protein kinase B. In addition, the activation of phospholipase C is most likely omitted, responsible for the release of GLUT4 vesicles on the cell membrane and for the regulation of oxidative status within the cell itself. There was also no significant difference in pAkt, attesting that the Liraglutide effects were not achieved through Akt activation. 

The combination of oxidative damage and high levels of glucose demonstrated the increasing effect of the pAKT/AKT ratio. Liraglutide treatment was effective only in higher dosages, implying that the metabolic effects of Liraglutide had to overcome oxidative damage first with secondary metabolic effects manifesting as the normalization of the pAKT/AKT ratios in the cells. Interestingly enough, the same effect as the one observed with high glucose and oxidative damage was produced when cells exposed to high glucose levels were treated with 20 nM of Liraglutide. Upon oxidative damage, the pAKT/AKT ratio increased, followed by an increase in ECM levels. Similar observations regarding the pAKT/AKT ratio and an increase in ECM levels were described by Palau et.al [41]. A significant increase in ECM levels treated with glucose and a high Liraglutide dose implied that Liraglutide itself in hyperglycemia could potentially induce kidney injury. Alternatively, in case of acute metabolic stress and increased oxidative damage, Liraglutide demonstrated beneficial effects by preventing the overproduction of ECM and decreased levels of pAKT/AKT to normal levels. Similar antioxidative effects were observed by Milani et.al. in a model of acute liver injury in mice [42].

Cells treated with HG30/H_2_O_2_ caused a reduction in the expression of the GSK3 beta protein. A significant increase was noted after the addition of Liraglutide at both concentrations to HG30 and HG30/H_2_O_2_ compared to HG30/H_2_O_2_-treated cells, supporting the hypothesis that the exposure to oxidative stress with high glucose levels triggers a lower insulin signaling pathway, independent of Akt affecting the GSK activity in tubule cells. It is also important to note that the fact that no fibrosis was measured in the cells treated with Liraglutide indicates that a hyperactivation of the beta catenin signaling pathway most likely did not occur [43]. Furthermore, pGSK3β represents the activity of GSK3β and was significantly increased after the treatment with HG30/Lira10nM compared to HG30/H_2_O_2_-exposed cells. Since kidney cells are capable of glycogen synthesis, the microenvironment could be the one responsible for a GSK3β transcription activation independent of the insulin induction. There was no difference between the control group and that of glucose-only-treated and glucose-treated groups in combination with both Liraglutide concentrations, suggesting that glucose alone is unable to induce changes in GSK3β expression levels. 

The levels of iNOS expression were significantly increased in cells treated with HG30/Lira10 compared to HG30/H_2_O_2_-treated cells. iNOS, a proinflammatory enzyme, has been shown to induce endothelial apoptosis in hyperglycemic conditions [44]. On the other hand, under physiological conditions, the production of NO by neuronal NOS (nNOS), endothelial NOS (eNOS) and inducible NOS (iNOS) has beneficial effects on vascular and cellular functions [45]. Conversely, in type-2 diabetes patients and nephropathy, a reduction of nitric oxide synthesis was observed [46]. In addition, high glucose and advanced glycosylation end products could cause a decrease in NOS expression in diabetic kidneys [47]. Moreover, iNOS dictates the levels of NO, which in turn has a wide range of cellular effects, and all of those effects are NO-concentration-dependent. Previous studies demonstrated that the upregulation of iNOS activity and NO can cause oxidative damage and cell death. On the other hand, short-term spikes in iNOS activity and NO levels act as a ROS-scavenging mechanism, demonstrated in various animal studies [48]. Therefore, the addition of Liraglutide 10 nM could restore NO production via iNOS stimulation compared to the extreme damage achieved by the combination of oxidative stress and hyperglycemia. 

pSTAT3 is directly related to the leptin signaling pathway. In the Western blot analysis of pSTAT3, we have observed the same pattern as for Akt activity. The treatment with HG30 /H_2_O_2_ /Lira10 increased pSTAT3 levels compared to all groups except control, indicating that oxidative stress, high glucose concentration and treatment with Liraglutide could lead to the activation of the leptin signaling pathway, which in turn will block part of the insulin pathway. These results suggest that this combination of high osmotic and oxidative stress and drugs could also have a potentially detrimental effect on cell metabolism due to a dysregulation of the insulin and leptin signaling pathways within cells. A research by Liu et al. reported that the acetylation and phosphorylation of p65 and STAT3 were higher in the glomeruli of db/db mice than in those of db/m mice [49]. Furthermore, a study by Opazo-Ríos L. et al. suggests an upregulation and compartmental activation of STAT1/3 in BTBR ob/ob diabetic kidneys, and a compensatory increase of SOCS1/3 proteins [20]. SOSCS3 levels remained unchanged, meaning that the segment of the leptin signaling pathway communicating with the insulin signaling pathway remained intact. This suggests that there was no pathological disruption of the metabolic network.

Increased ROS production by NOX4 in hyperglycemic conditions accompanied by the activation of TGF-ß promotes tubular damage [50]. Moreover, enhanced ROS production via Nox4 is necessary for fibronectin expansion and a high TGF-ß expression in tubular cells exposed to high glucose [51]. The effect of Liraglutide on the reduction of oxidative stress markers was confirmed by Hendarto, H. et al., demonstrating its beneficial role in the expression of NAD(P)H oxidase in the renal tissue (Nox4, gp91phox, p22phox, p47phox) of streptozotocin-induced diabetic rats, independent of antihyperglycemic effects [29]. In our study, cell exposure to HG30/H_2_O_2_ caused a significant decrease of NOX4 compared to cells treated only with HG30. In addition, a significant increase was observed in cells treated with HG30/H_2_O_2_/Lira10 compared to the HG30/H_2_O_2_. The level of oxidative stress induced in this study could have affected ROS production to the extent that the addition of the drug further potentiated cell injury and the toxic effect achieved by adding both, glucose and hydrogen peroxide. Furthermore, in cells treated only with glucose, without hydrogen peroxide, and with the addition of Liraglutide, no change in Nox4 expression has been observed, further supporting this notion. 

The major structural solute component in proximal tubule is F-actin [52]. Previous studies demonstrated that diabetes causes alterations in the cellular matrix, such as fibrosis and changes in the F-actin content and organization [53,54,55]. Hydrogen peroxide during inflammation causes cell death mainly by disarranging filamentous (polymerized) actin (F-actin). In a study by DalleDonne et al., the escalation in H_2_0_2_ concentration used for the treatment of actin monomers enhanced the number of actin filaments at a steady state [56]. Furthermore, in an in vitro study in mouse podocytes, high glucose led to filamentous actin (F-actin) rearrangement and injury [57]. Several studies have shown a more disorganized F-actin structure, with a decreased F-actin content in diabetic animals [58]. Correspondingly, a significant reduction in the distribution of F-actin was observed in cells treated with HG30/H_2_O_2_, but this effect was absent in cells exposed to hyperglycemia alone. Obviously, oxidative stress is the key element in F-actin filament disruption. 

While some studies have quantified F-actin fluorescence in diabetic animals, none have quantified F-actin disorganization at present. Previous data on exendin-4 treatment demonstrated a significant increase in the rearrangement of filamentous-actin with a reduction of cell migration in the cardiac and skeletal muscles of a mouse model [59]. Quite unexpectedly, the addition of Liraglutide to HG30 caused a significant decline of F-actin distribution, while no effect was detected in HG30/H_2_O_2_-treated cells. We can only speculate that the reduced distribution of F-actin with the addition of Liraglutide in hyperglycemic conditions is the result of actin filaments reorganization, increased differentiation and reduced cell migration, as with the addition of oxidative stress Liraglutide’s effect on the F-actin structure is less pronounced. 

The main limitation of our study is the lack of in vivo experiments confirming our in vitro findings. Therefore, the results of our study should be interpreted with discretion. In vivo studies are needed to further substantiate the data presented in this study. 

The results attained in this study support a possible protective role of Liraglutide in LLC-PK cells treated with hyperglycemia with and without H_2_O_2_, probably mediated via the inhibition of TGF-β1, thus reducing oxidative stress injury. Surprisingly, a WB analysis demonstrated that Liraglutide could play a dual role depending on the stress factor present. For instance, Liraglutide in combination with hyperglycemia could potentially induce kidney injury by the activation of pAkt/AKT levels. Furthermore, an increase in pSTAT3 and NOX4 levels stimulated by oxidative stress, high glucose concentration and Liraglutide suggested a potentially detrimental effect on cell metabolism due to the dysregulation of the insulin and leptin signaling pathways and the increased ROS production. On the other hand, the addition of Liraglutide to cells exposed to hyperglycemia and oxidative stress caused a decrease in pAKT/AKT levels, preventing ECM overproduction, and restored NO production via iNOS-minimizing oxidative stress damage. Another important finding shown by WB analysis was that Liraglutide’s effects were not achieved through the Akt activation of the upper insulin signaling pathway, but through an increase in GSK3β protein expression after the Liraglutide treatment indicated a possible activation of the lower insulin signaling pathway independent of Akt. The cellular model may not fully represent the results that could be obtained in animal experiments, but they are certainly a guideline and provide effective strategies for further in vivo research and clinical trials.

## Figures and Tables

**Table 1:** List of primary antibodies, secondary antibodies and tertiary complex used for the Western blot method.

Antibody Label	Full Name of the Antibody	Antibody Classification	Host Species	Manufacturer and Catalog Number	Dilution Used for Western Blot
Akt	Anti-protein kinase B	IgG monoclonal	Mouse	Cell signaling, Danvers, MA, USA Cat. No. 2920S	1:2000
pAkt	Anti-protein kinase B—phosphorylated on serine 473	IgG monoclonal	Rabbit	Cell signaling, Danvers, MA, USA Cat. No. 9271S	1:2000
GSK3 β	Anti-glycogen synthase kinase 3 alpha + beta	IgG monoclonal	Rabbit	Cell signaling, Danvers, MA, USA Cat. No. 5676S	1:2000
pGSK3 β	Anti-GSK3 beta—phosphorylated on tyrosine 216 + GSK3 alpha—phosphorylated on tyrosine 279	IgG polyclonal	Rabbit	Abcam, Cambridge, UK Cat. No. ab75745	1:2000
SOCS3	Anti-suppressor of cytokine signaling-3	IgG	Mouse	Abcam, Cambridge, UK Cat. No. ab14939	1:1000
pSTAT3	Anti-signal transducer and activator of transcription 3—phosphorylated on tyrosine 705	IgG monoclonal	Rabbit	Cell signaling, Danvers, MA, USA Cat. No. 9145S	1:2000
NOX4	Anti-NADPH oxidase 4	IgG	Goat	Santa Cruz Biotechnology, Dallas, TX, USA Cat. No. 21860	1:1000
iNOS	Anti-inducible nitric oxide synthase	IgG	Rabbit	Novus Biologicals, Littleton, CO, USA Cat. No. NB300-605	1:1000
αGO-biotin	Anti-goat antibody labeled with biotin	IgG	Donkey	Abcam, Cambridge, UK Cat. No. ab6884	1:20,000
αMO-biotin	Anti-mouse antibody labeled with biotin	IgG	Goat	Jackson Immunoresearch, West Grove, PA, USA Cat. No. 115-065-071	1:20,000
αRB-HRP	Anti-rabbit antibody labeled with HRP	IgG	Goat	Jackson Immunoresearch, West Grove, PA, USA Cat. No. 111-035-144	1:20,000
SA-HRP	Streptavidin peroxidase polymer, Ultrasensitive	-	-	Sigma-Aldrich, St. Louis, MO, USA Cat. No. S2438	1:1000

## Data Availability

The data presented in this study are available on request from the corresponding authors.
